# Control of Gene Expression by the Retinoic Acid-Related Orphan Receptor Alpha in HepG2 Human Hepatoma Cells

**DOI:** 10.1371/journal.pone.0022545

**Published:** 2011-07-26

**Authors:** Caroline Chauvet, Amandine Vanhoutteghem, Christian Duhem, Gaëlle Saint-Auret, Brigitte Bois-Joyeux, Philippe Djian, Bart Staels, Jean-Louis Danan

**Affiliations:** 1 Laboratoire de Pharmacologie, Toxicologie et Signalisation Cellulaire, INSERM UMR-S-747, Centre Universitaire des Saints Pères, Université Paris Descartes, Paris, France; 2 CNRS FRE-3210, Centre Necker, Université Paris Descartes, Paris, France; 3 CNRS FRE-3235, Centre Universitaire des Saints Pères, Université Paris Descartes, Paris, France; 4 Université Lille Nord de France, Lille, France; 5 INSERM, U1011, Lille, France; 6 UDSL, Lille, France; 7 Institut Pasteur de Lille, Lille, France; 8 Faculté de Médecine et de Pharmacie, INSERM U-905, Rouen, France; Ecole Normale Supérieure de Lyon, France

## Abstract

Retinoic acid-related Orphan Receptor alpha (RORα; NR1F1) is a widely distributed nuclear receptor involved in several (patho)physiological functions including lipid metabolism, inflammation, angiogenesis, and circadian rhythm. To better understand the role of this nuclear receptor in liver, we aimed at displaying genes controlled by RORα in liver cells by generating HepG2 human hepatoma cells stably over-expressing RORα. Genes whose expression was altered in these cells versus control cells were displayed using micro-arrays followed by qRT-PCR analysis. Expression of these genes was also altered in cells in which RORα was transiently over-expressed after adenoviral infection. A number of the genes found were involved in known pathways controlled by RORα, for instance *LPA*, *NR1D2* and *ADIPOQ* in lipid metabolism, *ADIPOQ* and *PLG* in inflammation, *PLG* in fibrinolysis and *NR1D2* and *NR1D1* in circadian rhythm. This study also revealed that genes such as *G6PC*, involved in glucose homeostasis, and *AGRP*, involved in the control of body weight, are also controlled by RORα. Lastly, *SPARC*, involved in cell growth and adhesion, and associated with liver carcinogenesis, was up-regulated by RORα. *SPARC* was found to be a new putative RORα target gene since it possesses, in its promoter, a functional RORE as evidenced by EMSAs and transfection experiments. Most of the other genes that we found regulated by RORα also contained putative ROREs in their regulatory regions. Chromatin immunoprecipitation (ChIP) confirmed that the ROREs present in the *SPARC*, *PLG*, *G6PC*, *NR1D2* and *AGRP* genes were occupied by RORα in HepG2 cells. Therefore these genes must now be considered as direct RORα targets. Our results open new routes on the roles of RORα in glucose metabolism and carcinogenesis within cells of hepatic origin.

## Introduction

Nuclear receptors are transcription factors, which are crucial in development and play major roles in metabolism and homeostasis (see [1] for review). Besides the classical nuclear receptors, which bind hormones and organic compounds, inverse genetic and genome sequencing approaches have identified a large number of nuclear receptor genes in the human and mouse genomes (see [2] for review). The function of some of these new receptors has been clarified in recent years and their ligands characterized. For others, however, their exact role is not understood and the nature of their ligand(s), if any, is still a matter of debate. Understanding in detail their function(s) is of major importance with potential crucial repercussions in the fields of metabolism, pharmacology and carcinogenesis.

Retinoic acid receptor-related Orphan Receptor α (RORα; NR1F1; [3], [4]) is one of these nuclear receptors. It has been cloned by virtue of its strong homology with the Retinoic Acid Receptor [5]. Most often RORα binds DNA as a monomer to specific sites called RORα response elements (ROREs). These elements consist of a 6-bp AT-rich sequence preceding the half-core PuGGTCA motif; they will be designated (A/T rich)_6_PuGGTCA in the rest of the manuscript [6]. The *Rora* gene generates four isoforms (RORα1–4), which differ in their N-terminal region [7]. Some putative ligands have been proposed for RORα: the natural hormone melatonin, synthetic thiazolidinediones and cholesterol derivatives, but some data are controversial and the nature of the RORα ligand(s) remains unclear [8], [9]. A spontaneous deletion within the *Rora* gene that prevents translation of the RORα ligand binding domain has been identified in the *staggerer* mouse (see [10] for review). Like the mice in which the *Rora* gene has been knocked-out by homologous recombination, the homozygous *staggerer* (*Rora^sg/sg^*) mutant mice suffer from severe cerebellar ataxia caused by massive neurodegeneration of Purkinje cells (see [10] for review). Moreover, analysis of the phenotype of the *Rora^sg/sg^* mouse has revealed the importance of RORα in regulating the inflammatory and immune responses [11], [12], in modulating atherosclerosis susceptibility [13], [14], and in post-ischemic angiogenesis [15].

RORα stimulates the transcription of the gene for the NFκB inhibitor IκB [11]. The gene encoding apolipoprotein A-I, a major component of high density lipoproteins, has also been shown to be controlled by RORα in rats [14]. We and others have provided evidence that RORα may participate in the control of α-fetoprotein [16], apolipoprotein C-III [17], apolipoprotein A-V [18], and fibrinogen-ß gene expression in liver [19]. Recent findings have indicated that the *Rora* gene is up-regulated by hypoxia *via* a Hypoxia Inducible Factor-1 (HIF-1)-dependent mechanism [20]. RORα also regulates HIF-1 at the transcriptional level [21]. These data have brought new information about the possible roles of RORα in (patho)physiological processes such as lipid metabolism, response to inflammation, angiogenesis and carcinogenesis where hypoxia plays an important role. The implication of RORα in these important functions has been recently demonstrated (see [22], [23] for reviews).

It is also known that RORα, together with another nuclear receptor Rev-erbα (NR1D1; [4]), participates in the control of the circadian clock and that there are interactions of physiological significance between the circadian clock and metabolism ([24] and see [22] for review).

Large-scale techniques, such as characterization of the transcriptome of cells or organs, are powerful tools to display genes controlled by a transcription factor. In the case of ROR, they have been used to identify genes differentially expressed in the liver of mice lacking functional RORα and RORγ proteins and in the liver of wild-type control mice. These studies have demonstrated that RORα and RORγ control genes involved in phase I and II metabolism [25].

In the present study we have used micro-arrays to analyze the modifications of the transcriptome of HepG2 human hepatoma cells upon stable over-expression of RORα. This approach, combined with electrophoretic mobility shift assays (EMSAs), reporter gene expression and chromatin immunoprecipitation (ChIP), revealed several novel hepatic RORα target genes with interest in physiology and pathophysiology.

## Results

### Over-expression of RORα1 alters gene expression in HepG2 human hepatoma cells

**Figure 1:**
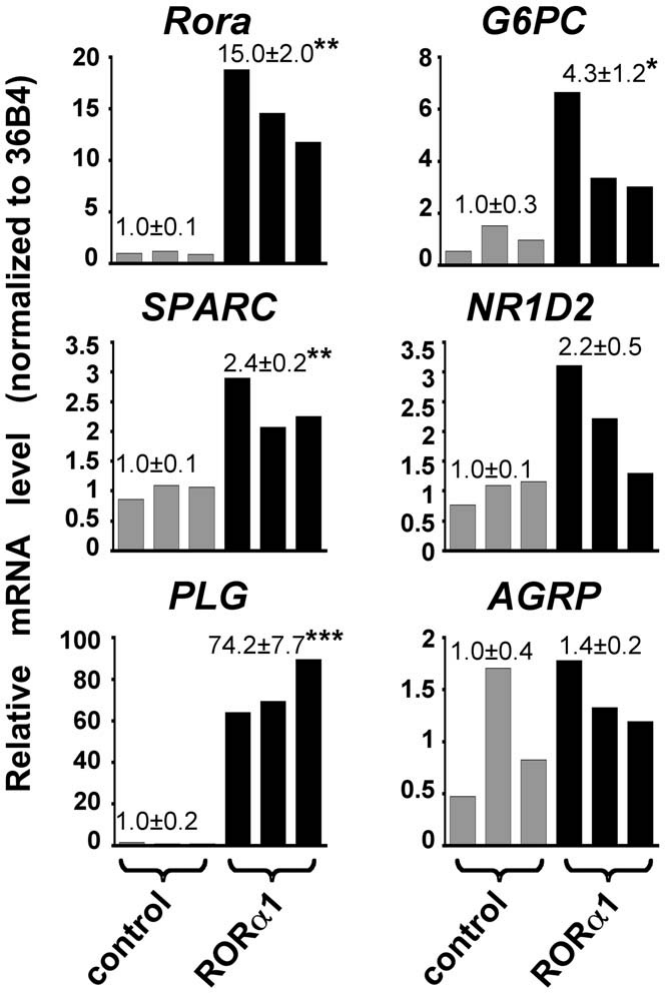
qRT-PCR analysis of expression of several of the genes found to be up-regulated in HepG2 cells over-expressing RORα upon micro-array analysis. Aliquots of the RNA samples from stably-transfected HepG2 cells over-expressing RORα1 and from stably transfected control cells that have been analyzed by the DNA micro-array technology were submitted to qRT-PCR analysis for measuring the levels of *Rora*, *SPARC*, *PLG*, *G6PC*, *NR1D2*, and *AGRP* mRNAs. The mRNA levels were normalized to the *36B4* mRNA level. Results corresponding to HepG2 cells over-expressing RORα1 (black bars) are given relatively to the control cells (grey bars). Values refer to the means +/− S.E.M. of the fold changes (n = 3 independent pools of transfected cells). *: *P*<0.05, **: *P*<0.01, ***: *P*<0.001 between RORα1 over-expressing and control cells.

We used a DNA micro-array technique, which we had previously validated by monitoring the effect of hypoxia on gene expression in HepG2 cells (data not shown) to display genes whose expression is controlled by RORα in HepG2 cells. We analyzed RNA prepared from HepG2 cells stably transfected with the pCMX-hRORα1 vector and over-expressing human RORα. HepG2 cells stably transfected with the pCMX empty vector were used as control. To ascertain that the stably transfected cells over-produced RORα, we first monitored by qRT-PCR the levels of *Rora* mRNA. The *Rora* mRNA levels were found to be largely increased (15.0+/−2.0 fold, n = 3, p<0.01) in pCMX-hRORα1 transfected cells (Fig. 1).

To confirm that the recombinant RORα protein is over-produced and functional in the stably transfected cells, we measured the mRNA levels of the Rev-erbα (NR1D1) nuclear receptor, whose gene is a known target of RORα [26], [27]. The level of *NR1D1* mRNA was significantly higher (2.1+/−0.2 fold, n = 3, p<0.01) in cells over-expressing RORα than in control cells. Taken together, these data indicated that HepG2 hepatoma cells stably transfected with the pCMX-hRORα1 vector over-produced biologically active RORα protein.

Large-scale analysis using DNA micro-arrays was then performed on RNA prepared from HepG2 cells stably over-expressing RORα and from control cells. The results showed that expression of a number of genes was altered by over-expression of RORα. Most of the modulated genes were assigned and are listed in Table 1. All genes but two were up-regulated. Sequences not assigned to a known gene are given in Supporting Information S2.

**Table 1:** Micro-array analysis of the effects of a stable RORα over-expression on gene expression in HepG2 cells.

Gene symbol	Gene description	Genbank accession	Feature Number	Fold change	Mean±SEM	*P*value
*Up-regulated*						
PLG	plasminogen	NM_000301	12618	25.8427.2429.65	*27.58±1.11*	6.40E-151.39E-192.15E-19
RORA	RAR-related orphan receptor A	NM_134260	31288	12.9816.4513.83	*14.42±1.04*	3.73E-207.29E-212.33E-20
LPA	lipoprotein, Lp(a)	NM_005577	31776	10.088.409.60	*9.36±0.5*	3.59E-121.02E-142.47E-16
FBXL17	F-box and leucine-rich repeat protein 17	BC018548	391	5.492.692.19	*3.46±1.03*	1.13E-089.20E-054.88E-05
G6PC	glucose-6-phosphatase, catalytic subunit	NM_000151	19594	2.783.032.61	*2.80±0.12*	6.73E-074.06E-073.26E-06
SPARC	secreted protein, acidic, cysteine-rich (osteonectin)	NM_003118	4259	2.842.232.03	*2.37±0.24*	1.13E-095.19E-075.64E-06
AGRP	agouti related protein homolog (mouse)	NM_001138	21543	2.742.661.44	*2.28±0.42*	1.89E-036.19E-041.34E-01
NR1D2	nuclear receptor subfamily 1, group D, member 2	BC015929	31530	2.082.911.68	*2.22±0.36*	3.04E-052.02E-081.86E-03
SMOC1	SPARC related modular calcium binding 1 (SMOC1)	NM_022137	32907	2.101.752.68	*2.18±0.27*	2.54E-062.09E-045.11E-09
HEPN1	associated with liver cancer	NM_001037558	34970	1.812.182.50	*2.16±0.20*	2.58E-031.52E-059.65E-07
MLLT7	myeloid/lymphoid or mixed-lineage leukemia translocated to 7	NM_005938	42837	1.912.002.22	*2.04±0.09*	4.52E-031.02E-039.14E-05
*Down-regulated*						
LGI2	leucine-rich repeat LGI family, member 2	NM_018176	24947	0.190.360.37	*0.31±0.06*	1.88E-054.57E-042.46E-03
ADIPOQ	adiponectin, C1Q and collagen domain containing	NM_004797	36194	0.340.170.17	*0.23±0.06*	3.21E-036.58E-044.51E-04

HepG2 cells were stably transfected with the pCMX-hRORα1 expression vector or with the pCMX insertless vector as a control. Total RNAs were extracted and mRNA levels were compared between these two experimental conditions with Agilent micro-arrays. This analysis revealed several genes whose expression was up-regulated or down-regulated by at least 2 fold in cells over-expressing RORα as compared to control cells. The results relative to sequences corresponding to known genes are shown. The gene symbols, gene description, genbank accession numbers and feature numbers on micro-arrays are given in columns 1 to 4. Gene expression levels in RORα over-expressing cells are expressed relatively to those in control cells. Results are given in column 5 as fold changes and in column 6 as the means of these fold changes +/− S.E.M. (n = 3 independent pools of transfected cells). *P*values generated by the micro-array analysis are shown in column 7. It refers to the statistical significance of the differences between cells over-expressing RORα and control cells (all *P*values are less than 0.01 except one over three for *AGRP*). Results corresponding to one spot/Feature Number representative of two are shown for *PLG*.

We checked the validity of our DNA micro-array analysis by measuring by qRT-PCR the mRNA levels of *SPARC*, *PLG*, *G6PC*, *NR1D2 (Rev-erbb)*, and *AGRP* on aliquots of the RNA samples used in the micro-array analysis. The qRT-PCR results showed a statistically significant increase in the mRNAs for *SPARC*, *PLG*, and *G6PC* in cells over-expressing RORα (Fig. 1) and thus confirmed the results of the micro-array analysis. Although results obtained for *NR1D2* and *AGRP* were not statistically significant, they showed a tendency towards an increase in RORα-over-expressing cells (Fig. 1). Our approaches thus displayed a subset of genes specifically controlled by RORα in HepG2 human hepatoma cells.

### Expression of the genes revealed in our micro-array analysis is stimulated in HepG2 cells infected with an adenovirus over-expressing RORα1

**Figure 2:**
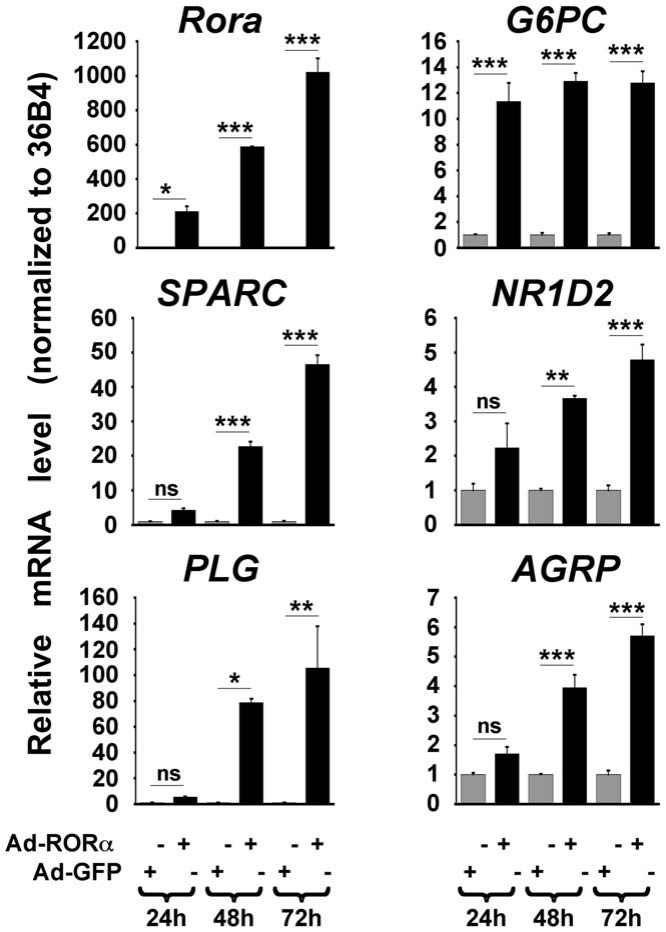
qRT-PCR analysis of expression of RORα-regulated genes in HepG2 cells over-expressing RORα1 following adenoviral infection. HepG2 cells were infected with recombinant adenoviral vectors allowing expression of either RORα1 or GFP as a control. Total RNAs were extracted at 24 h, 48 h and 72 h following adenoviral infection. Aliquots were then analyzed by qRT-PCR for measuring the levels of *Rora*, *SPARC*, *PLG*, *G6PC*, *NR1D2*, and *AGRP* mRNAs. The mRNA levels were normalized to the *36B4* mRNA level. Results corresponding to HepG2 cells over-expressing RORα1 (black bars) are given relatively to the control cells over-expressing GFP (grey bars). Values refer to the means +/− S.E.M of the fold changes (n = 3 independent experiments). ns: non statistically significant, *: *P*<0.05, **: *P*<0.01, ***: *P*<0.001 between RORα1 over-expressing and control cells.

In order to avoid possible artifacts inherent in the use of stably modified cultured cells over-expressing a transcription factor, we thought important to further check, by a complementary approach, the validity of the results obtained in stable transfectants. We infected HepG2 cells with an adenoviral vector expressing the RORα1 cDNA (Ad-RORα), and monitored by qRT-PCR the expression of several of the genes identified in stably transfected cells. As controls, HepG2 cells were infected with an adenoviral vector expressing the Green Fluorescent Protein (Ad-GFP) cDNA. The levels of the *Rora*, *SPARC*, *PLG*, *G6PC*, *NR1D2* and *AGRP* mRNAs were measured by qRT-PCR 24 h, 48 h and 72 h after infection of HepG2 cells with either Ad-RORα or control Ad-GFP. These experiments showed that the expression of all the analyzed genes including *NR1D2* and *AGRP* was significantly up-regulated in response to adenoviral-mediated over-production of RORα in HepG2 cells (Fig. 2). The level of *NR1D1* mRNA was also increased in response to adenoviral-mediated over-expression of RORα (data not shown).

In summary, it appears that our results were generally corroborated in two cellular models (stably-transfected and adenovirus-infected cells), and using two techniques for measuring gene expression: DNA micro-arrays and qRT-PCR. Therefore our results converge to show that expression of the subset of genes listed in Table 1 is controlled by RORα in HepG2 cells. Although the expression of the genes for NR1D2 and AGRP was not significantly affected by RORα1 over-expression in stable transfectants, because of sample variability (Fig. 1), the effect of RORα1 on these two genes became clearly visible after adenovirus-mediated over-expression (Fig. 2).

### The promoter of the human *SPARC* gene contains a functional RORE

Our present study points to several novel genes as being regulated, at least in part, by RORα in cells of hepatic origin. However, it is important to know whether these genes are direct or indirect targets for RORα.

In this context, and because of the crucial importance of SPARC in several cell functions in different organs, including liver, both during normal life and in carcinogenesis (see [28] for review), we chose to test if the *SPARC* gene was directly regulated by RORα. We detected one putative RORE (5′-TGTTCTGGGTCA-3′) between nucleotides −285 and −274 relative to the transcription start site in the human *SPARC* promoter [29]. We searched if this putative RORE was conserved in other species and we found that this element is totally conserved in Chimpanzee, Orangutan and partially conserved (5′-TGTTCTAGGTGA-3′) in Mouse, Rat, and Horse. We investigated whether this putative RORE was functional in terms of RORα binding and transcriptional control in HepG2 cells and if consequently, the *SPARC* gene was susceptible to be directly controlled by RORα.

**Figure 3:**
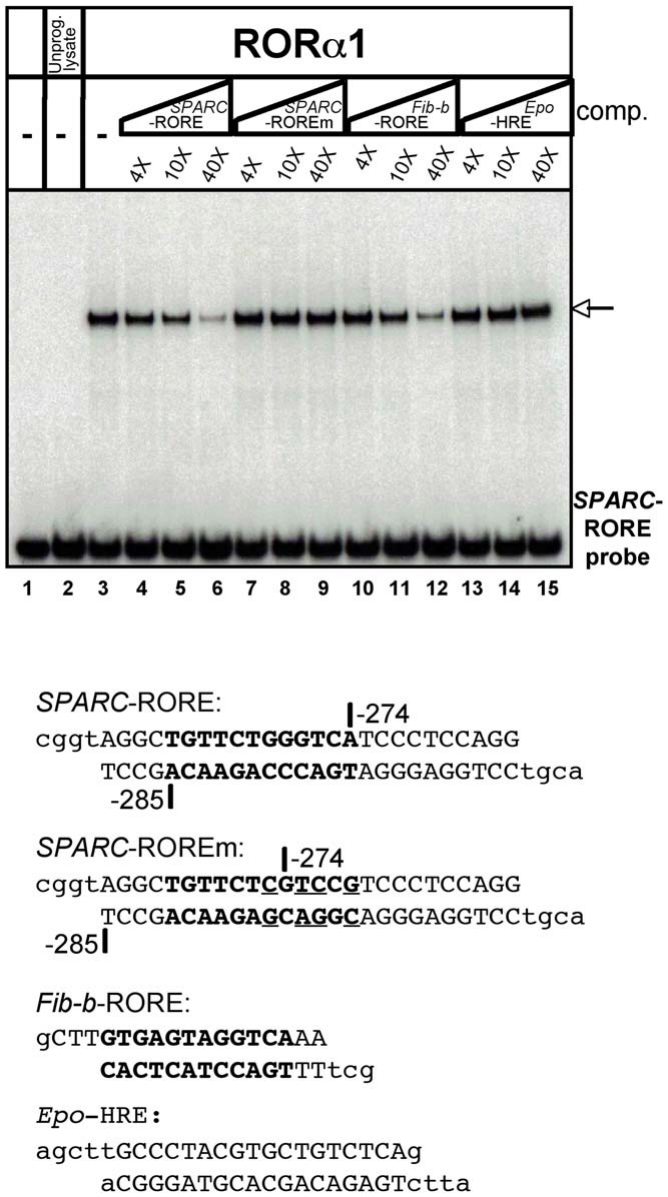
RORα binds to a RORE in the promoter of the human *SPARC* gene. EMSAs were performed by using a radiolabeled double-stranded oligonucleotide containing the DNA sequence corresponding to region −289 to −264 of the human *SPARC* promoter (*SPARC*-RORE probe). The probe was incubated alone or with 1 µl of unprogrammed reticulocyte lysate as controls (lanes 1 and 2, respectively), or with 1 µl of RORα1-programmed lysate (lanes 3–15). Competition assays were carried out by incubating the *SPARC*-RORE probe with the RORα1-programmed lysate in the presence of unlabeled double-stranded oligonucleotides corresponding to the wild-type *SPARC*-RORE (lanes 4–6), or the mutated *SPARC*-ROREm (lanes 7–9), or the RORE of the human *fibrinogen-ß* gene (lanes 10–12), or the hypoxia response element of the *EPO* gene (lanes 13–15) at a 4-, 10-, or 40-fold molar excess. The arrow points to the complex formed upon binding of RORα to the *SPARC*-RORE probe. Sequences of the oligonucleotides are given in the lower part of the figure. The RORE sequence is written in bold. Nucleotides written in lowercase were added for convenience.

We first performed EMSAs to determine if the RORα protein bound to the putative SPARC-RORE. As shown in Fig. 3, a complex was formed between RORα and the labeled oligonucleotide. Specificity of binding was confirmed by competition experiments using unlabelled oligonucleotides covering the wild-type or mutated SPARC-RORE, the *fibrinogen-ß*-RORE [19], and an unrelated oligonucleotide containing the Hypoxia Response Element of the *EPO* gene [20]. These experiments clearly demonstrated that RORα specifically bound to the DNA element present between −285 and −274 in the promoter of the human *SPARC* gene.

**Figure 4:**
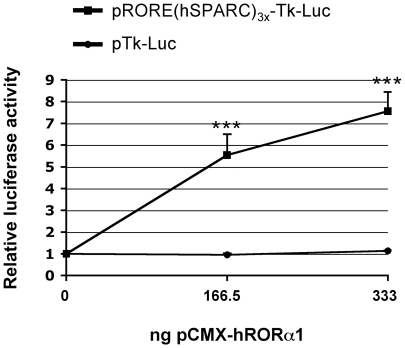
RORα activates transcription through the (−285/−274) RORE of the *SPARC* promoter in HepG2 cells. HepG2 cells were transiently cotransfected in 12-well plates with 666 ng/well of the pRORE(hSPARC)_3x_-Tk-Luc luciferase reporter vector or with 666 ng/well of the pTk-Luc control luciferase reporter vector and with increasing amounts (0-, 166.5-, or 333-ng/well) of pCMX-hRORα1 expression vector. The systematic cotransfection with 83 ng/well of the pRL-Tk normalization vector allowed the normalization of the luciferase activities obtained with the reporter vectors. The total amount of transfected DNA was kept constant to 1082 ng/well by adding the pCMX insertless vector. The normalized activities of the vectors in the presence of pCMX-hRORα1 were expressed relatively to that in the absence of this expression vector. Results are given as means +/− S.E.M. (n = 6 independent experiments). ***: *P*<0.001 between cells transfected with pRORE(hSPARC)3x-Tk-Luc and cells transfected with pTk-Luc.

To determine if the SPARC-RORE was functionally active, we carried out transient transfections in HepG2 cells with plasmids containing a luciferase reporter gene placed under the control of the herpes simplex virus thymidine kinase (Tk) promoter either alone or in association with three tandem copies of the SPARC-RORE. Co-transfection of these plasmids with a vector overexpressing the RORα cDNA showed that presence of the three ROREs resulted in a large increase in RORα-induced luciferase activity (Fig. 4). It may be concluded that RORα activates transcription upon binding to the RORE element present in the *SPARC* gene promoter.

Taken together, the results from the EMSAs and the transient tranfections showed that the SPARC-RORE is functionally active in HepG2 cells. They indicated that the *SPARC* gene is, at least in part, under the direct transcriptional control of RORα. Consequently *SPARC* must now be considered as a RORα target gene in HepG2 cells.

### The regulatory regions of most of the genes disclosed in the micro-array analysis contain putative ROREs

To determine whether some of the other human genes displayed in our micro-arrays were putative direct RORα target genes, we searched their regulatory regions (promoter and intragenic regions) for DNA sequences resembling the (A/T rich)_6_PuGGTCA motif characteristic of ROREs and found in the *SPARC* promoter. For this purpose we used the Genomatix-based matrices described in Materials and Methods. This analysis was carried out on ten of the human genes that we had identified (*PLG*, *FBXL17*, *G6PC*, *AGRP*, *NR1D2*, *SMOC1*, *HEPN1*, *MLLT7*, *LGI2*, *ADIPOQ*). We found that the regulatory regions of nine out of these ten genes contained at least one DNA sequence matching the RORE consensus (Table 2). Importantly, several of these putative ROREs are also conserved in at least one species other than the Human (Table 2 and Supporting Information S3). This evolutionary conservation favors the hypothesis that the putative ROREs are physiologically relevant. Search of the whole human genome for ROREs using Genomatix-based matrices revealed a frequency of 1.41 ROREs per 1000 bp. In view of this high frequency, it appeared necessary to determine whether the genes displayed in our micro-arrays were indeed direct RORα targets *in vivo*.

**Table 2:** Identification of putative ROREs in the genes displayed by the micro-array analysis.

Gene symbol	Putative RORE sequence	Position in the human gene	RORE conservation in other species
PLG	tc**tcatgtAAGTCA**acaacatcc	from −6 to +17 (promoter)	Chimpanzee, Rhesus macaque, Mouse, Rat
FBXL17	at**tattgtAAGTCA**ccgtaagag	intragenic (intron 2)	Chimpanzee
FBXL17	ct**aaacaaAGGTCA**cgccagtac	intragenic (intron 2)	Chimpanzee, Rat, Dog, Chicken
G6PC	ca**gtattcAGGTCA**acccagccc	from −66 to −44 (promoter)	Mouse, Rat, Dog
AGRP	aa**gaattcGGGTCA**aggacattg	from −140 to −118 (promoter)	Chimpanzee, Rat, Dog, Cow
NR1D2	tg**gctcttAGGTCA**aagcggtgg	from −388 to −366 (promoter)	Rhesus macaque, Dog
NR1D2	gc**atgaccAGGTCA**accttttaa	intragenic (intron 5)	Chimpanzee, Rhesus macaque, Mouse, Dog, Cow, Zebrafish
HEPN1	gc**ataactATGTCA**ggaagacag	from −260 to −238 (promoter)	-
MLLT7	tc**tcaatcTGGTCA**cctacacga	from −944 to −922 (promoter)	Chimpanzee, Rhesus macaque, Dog
LGI2	ca**tgacttTGGTCA**agtcactta	intragenic (intron 2)	Chimpanzee, Dog, Horse
LGI2	ag**aaaatcTGGTCA**aaaggaatc	intragenic (intron 2)	Chimpanzee, Rhesus macaque
ADIPOQ	ca**aaataaGGGTCA**aggcctgga	from −243 to −221 (promoter)	Chimpanzee
ADIPOQ	ca**ctgagtTGGCCA**atgggaaat	from −93 to −71 (promoter)	Chimpanzee, Mouse

Regulatory regions (600–1100 bp promoters or intragenic regions) of ten human genes displayed by our over-expression experiments were extracted from sequence databases (Genomatix, UCSC, Genbank) and analyzed for the presence of sequences close to the RORE consensus ((A/T rich)_6_PuGGTCA). We found that regulatory regions of nine human genes out of ten contain one or two putative ROREs. The gene symbols are given in column 1. Sequences containing the putative ROREs (in bold) are in column 2. Positions of these sequences relatively to the transcriptional start sites (TSS; +1) in the human promoters or positions in the intragenic regions are in column 3. Some promoters contain three possible TSSs (*PLG*, *G6PC*, *NR1D2*, *MLLT7*, *ADIPOQ*). In these cases, the positions of the sequences are relative to the downstream TSS. Species in which the RORE is totally or partially conserved are given in column 4 (the RORE sequences found conserved in the species available in the databases are shown in Supporting Information S3). No RORE has been found in the human *SMOC1* promoter. The human *LPA* promoter could not be analyzed because it was not referenced in the Genomatix program.

### Chromatin immunoprecipitation demonstrates that RORα binds *in vivo* to the regulatory regions of the genes identified in the micro-array analysis

**Figure 5:**
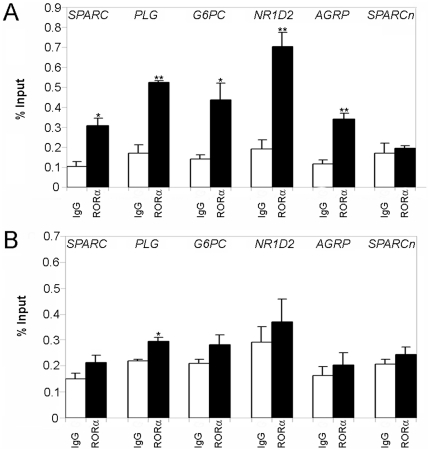
qPCR analysis of immunoprecipitated chromatin shows that RORα binds to the regulatory regions of *SPARC*, *PLG*, *G6PC*, *NR1D2* and *AGRP* in HepG2 cells. Chromatin was prepared from stably-transfected HepG2 cells over-producing RORα1 (A) or not (B) and fragmented by sonication. Chromatin fragments were then immunoprecipitated with a specific anti-RORα antibody (black bars) or with a nonspecific IgG as a negative control (white bars). qPCR (normalized to input) was used to amplify genomic fragments covering the ROREs identified *in silico* in the *SPARC*, *PLG*, *G6PC*, *NR1D2* (intron 5) and *AGRP* genes. qPCR amplification of a DNA region located in the *SPARC* gene but devoid of RORE was used as a negative control (*SPARCn*). Data are represented as the means +/− S.E.M. (n = 3). *: *P*<0.05, **: *P*<0.01 between samples treated with the anti-RORα antibody and samples treated with nonspecific IgG.

We carried out ChIP in HepG2 cells using an anti-RORα antibody. Immunoprecipitated DNA fragments were submitted to qPCR using primers specific for *SPARC*, *PLG*, *G6PC*, *NR1D2* and *AGRP*. The regions amplified contained the ROREs that we had identified using the Genomatix matrices. Results are shown in Fig. 5. For all five genes examined, HepG2 cells stably over-producing RORα1 yielded a statistically significant ChIP enrichment when the anti-RORα specific antibody was used as compared to the nonspecific IgG (Fig. 5A). No difference was detected in HepG2 cells producing normal amounts of RORα for *SPARC*, *G6PC*, *NR1D2* and *AGRP* (Fig. 5B). The *PLG* gene alone showed a statistically significant enrichment (Fig. 5B). The specificity of the ChIP was further demonstrated by the fact that an irrelevant region located 9300 bp downstream of the transcription start site in the human *SPARC* promoter [29] was not enriched by immunoprecipitation with the anti-RORα antibody (Fig. 5). Taken together, our data show that the ROREs that we identified in the *SPARC*, *PLG*, *G6PC*, *NR1D2* and *AGRP* genes can bind RORα *in vivo* in the context of HepG2 cells over-expressing RORα. Therefore, these genes must be considered as novel targets for RORα.

## Discussion

Very little is known on the biological function of RORα and its target genes in cells of hepatic origin. To our knowledge, the only previous such study of RORα target genes is a micro-array analysis comparing the liver of RORα-mutant *staggerer* mice with that of wild-type mice [25]. Because RORα is widely expressed, these mice exhibit a broad spectrum of physiological defects, such as abnormalities in eating behavior, inability to maintain correct body temperature, to move properly, etc… Therefore, when analyzing the liver phenotype of the *staggerer* mice, it is difficult to sort out the direct effects of the mutation on liver cells from indirect ones that may find their origin outside of the liver. Such difficulty does not exist when examining cultured human hepatoma cells, where the effect of RORα on gene expression is necessarily direct. Therefore in order to increase our knowledge on RORα function in human hepatic cells, we analyzed, using DNA micro-arrays, the changes in the expression of genes induced by a stable over-expression of this transcription factor in HepG2 human hepatoma cells. We thus identified *SPARC*, *PLG*, *G6PC*, *NR1D2* and *AGRP* as RORα targets in hepatoma cells. The results obtained with the micro-arrays were confirmed by qRT-PCR. We cannot however exclude that our approach did not display all the RORα-sensitive genes present in HepG2 cells. Interestingly, the genes whose expression was modulated in cells stably over-expressing RORα also showed altered expression in cells in which RORα was transiently over-expressed following infection with a RORα adenoviral vector. The convergence of these two approaches strengthened the conclusion that RORα modulates the expression of the genes that we identified. The fact that the regulatory region of most, if not all of these genes, possessed a DNA sequence very close to the RORE consensus lent further support to the idea that they are indeed RORα target genes. This hypothesis was confirmed by ChIP experiments showing that the ROREs were occupied by RORα in the cells. In addition, we showed that in the case of the *SPARC* promoter, binding of RORα led to transactivation in HepG2 cells. However, we could not detect a correlation between the level of chromatin occupancy by RORα and its target gene expression. We may hypothesize that RORα affects transcription both directly and indirectly, possibly by changing protein-protein interaction networks.

We showed that expression of the *Rev-erba* gene is higher in HepG2 cells in which RORα is over-expressed. Rev-erbα (NR1D1) is a nuclear receptor, whose gene is known to be a direct target for RORα [26], [27]. This strongly reinforces our findings. It is then reasonable to think that the genes listed in Table 1 are indeed modulated by RORα in hepatic cells. Some of the RORα-modulated genes are involved in physiological functions such as lipid metabolism (*LPA*, *NR1D2*, *ADIPOQ* also known as *adiponectin*) and inflammation (*ADIPOQ*, *PLG*), but also in related cardiovascular diseases such as atherosclerosis [30], [31], [32], [33]. Interestingly, it is well known that RORα plays a role in these functions (see [23] for review). Moreover, *PLG*, another RORα target gene displayed by the present study, is involved in fibrinolysis, wound healing, tumor growth and metastasis [34]. Interestingly, PAI-1, another component of the fibrinolytic system, and fibrinogen-ß, one of the chains of the coagulation factor fibrinogen, are also RORα targets [19], [35]. These data strongly suggest that RORα is involved in the fine control of coagulation/fibrinolysis with potential impacts on processes such as wound healing, tumor growth, metastasis and atherothrombosis.

Our results call attention to new metabolic pathways or patho-physiological states in which RORα may play an important regulatory role. We show that RORα governs expression of two other monomeric nuclear receptors of the Rev-erb family. In addition to *Rev-erba* known to be a direct RORα target gene [26], [27], we show here that expression of the NR1D2 nuclear receptor, also known as Rev-erbß, is also controlled by RORα. This finding is of importance in the field of chronobiology since RORs and Rev-erbs nuclear receptors constitute essential players in the circadian clock ([24] and see [22] for review). The interrelationship between RORs and Rev-erbs nuclear receptors susceptible to bind to similar DNA target sequences could tightly control enzymes involved in liver metabolism and whose expression/activity is known to oscillate as a function of time throughout the day. In these respects, our finding that Rev-erbß expression is, at least in part, controlled by RORα adds another degree to the complexity and consequently to the finely tuned regulation by the mammalian clock of enzymes and transport proteins synthesized by the liver, and whose various functions include lipid metabolism, coagulation/fibrinolysis, etc….

We and others have shown that RORα controls genes encoding enzymes or transport proteins such as α-fetoprotein [16], ApoA-I [14], ApoC-III [17], and ApoA-V [18], all of which play key roles in lipid metabolism/transport. It has also been shown recently that RORα controls adipocyte differentiation [36]. Our present data showing that the expression of *G6PC*, which encodes a subunit of G6Pase, is controlled by RORα in HepG2 cells are in agreement with a study showing that *G6PC* is a RORα target gene in mouse liver [37]. They agree with the fact that G6Pase is regulated by Rev-erbα [38]. Our results support the notion that RORα may play a role in controlling the carbohydrate metabolism, besides its function in lipid metabolism. G6Pase is a key enzyme in the regulation of hepatic glucose production and blood glucose homeostasis. It catalyzes the terminal step in the glycogenolytic and gluconeogenic pathways (see [39] for review). Mutations in the *G6PC* gene result in glycogen storage disease type 1a mainly characterized by hypoglycemia [40]. Tight links between G6Pase and the insulin regulatory pathway have been reported (see [39] for review). *ADIPOQ*, which encodes a protein also involved in both lipid and glucose metabolism has also been identified as a RORα target in this study [41]. That RORα and HIF-1α regulate each other under hypoxic stress [20], [21] further supports a role for RORα in carbohydrate metabolism since it is well known that the carbohydrate metabolism pathway is greatly altered during the hypoxic stress (see [42] for review). All these data argue in favor of a new role for RORα in controlling carbohydrate metabolism.

We show that the *SPARC* gene, also known as *Osteonectin* is a new RORα target gene. This gene encodes a matricellular protein that controls cell-cell and cell-matrix adhesion. It may also play a role in cell growth and, depending on the cell type, this multi-faceted protein is associated with a highly aggressive tumor phenotype or behaves as a tumor suppressor (see [28] for review). In hepatocellular carcinomas, SPARC expression correlates with tumor angionenesis [43]. Expression of the *SPARC* gene is up-regulated in fibrotic liver [44] while its down regulation by adenoviral expression of an antisense SPARC attenuates liver fibrosis [45]. Interestingly, SPARC over-expression in hepatocellular carcinoma cells results in a reduced tumorigenicity [46]. Another gene that we found up-regulated by RORα is *HEPN1*, which encodes a peptide involved in the control of cell growth and apoptosis, and whose expression is down-regulated in hepatocellular carcinoma [47]. These data strongly suggest a role for RORα at different steps of liver carcinogenesis.

In conclusion, our results point to possible roles of the RORα nuclear receptor in liver cells (lipid and carbohydrate metabolism, circadian clock, inflammation and carcinogenesis). They also open new routes to think about a more general role for RORα in (patho)physiology.

## Materials and Methods

### Cell culture

HepG2 human hepatoma cells (American Type Culture Collection HB-8065) were cultured as monolayers at 37°C in a humidified 95% air/5% CO_2_ incubator. The culture medium used was a 1∶1 mixture of Dulbecco's Modified Eagle's Medium and Ham-F12 medium with Glutamax-I, supplemented with 10% (v/v) fetal bovine serum, 100 µg/ml gentamycin, and 2.5 µg/ml fungizone (all from Invitrogen). HepG2 cells were 70–80% confluent before use.

### Vectors, stable and transient transfections and luciferase assay

The pCMX-hRORα1 expression vector containing the human RORα1 cDNA and the pCMX empty vector were from Dr. V. Giguère [7]. The pPGK-neo expression vector contains the phosphoglycerate kinase 1 promoter driving the neomycin phosphotransferase gene [48]. The pRORE(hSPARC)_3x_-Tk-Luc was obtained by cloning three copies (sense-sense-sense) of the RORE, located between nucleotides −274 and −285 of the human *SPARC* promoter in the *Bgl*II restriction site in the polylinker of the TkpGL3 firefly luciferase reporter vector (referred to as pTk-Luc in the rest of this manuscript and previously described in [49]). To this aim, the oligonucleotides hSPARC-RORE_3x_/U, 5′-GATCTGCTGTTCTGGGTCATCCCGCTGTTCTGGGTCATCCCGCTGTTCTGGGTCATCCCA-3′, hSPARC-RORE_3x_/L, 5′-GATCTGGGATGACCCAGAACAGCGGGATGACCCAGAACAGCGGGATGACCCAGAACAGCA-3′were used. Identity of the pRORE(hSPARC)_3x_-Tk-Luc vector was verified by DNA sequencing. The pRL-Tk vector (Promega) contains the herpes simplex virus thymidine kinase promoter driving the renilla luciferase reporter gene. Stable transfection experiments were done using the calcium phosphate precipitation method exactly as described [19]. Transient transfection experiments were done using the Fugene 6 reagent (Roche Molecular Biochemicals). Cells cultured in 12-well plates were co-transfected with 666 ng/well of pTk-Luc or pRORE(hSPARC)_3x_-Tk-Luc reporter vector with 0-, 166.5- or 333-ng/well of pCMX-hRORα1 expression vector. 83 ng of pRL-Tk expression vector was added in each well in order to normalize transfection efficiencies. The total amount of transfected DNA was kept constant to 1082 ng/well by addition of the pCMX insertless vector. The cells were lysed 48 h after transfection in 200 µl of reporter lysis buffer (Promega). Luciferase activity was assayed using the Dual-Luciferase Reporter Assay System (Promega). The firefly luciferase activity was normalized with the renilla luciferase activity.

### Adenovirus infection

Recombinant Ad-GFP and Ad-RORα1 were obtained by homologous recombination in *Escherichia coli* [50] after insertion of the cDNAs into the pAdCMV2 vector. Viral stocks were titrated as previously described [51]. HepG2 cells were infected overnight at an input multiplicity of 100 virus particles per cell by adding virus stocks directly to the culture medium. Cells were harvested 24, 48, 72 hours after the end of the infection for RNA preparation.

### Micro-array analysis

Total RNA was isolated using TRIZOL™LS reagent (Invitrogen). Preparation of the labeled cDNA samples, hybridization of oligonucleotide microarrays and data analyzes were performed by the Diagnogene society (Diagnogene, division of Imaxio, Saint-Beauzire, France) using the system developed by Agilent Technology. Briefly, quality and quantity of the total RNA samples were assessed by measuring the UV absorbance at 260 and 280 nm with a spectrophotometer and performing an electropherogram with 2100 Bioanalyzer (Agilent Technology). Then 500 ng of total RNA was used to synthesize fluorescently labeled cRNA probes by incorporation of cyanine 3- or cyanine 5-CTP using the low input linear amplification kit (Agilent Technology), according to the manufacturer's guidelines. Labeled cRNA samples were purified with RNeasy mini spin columns (Qiagen) and the quantity and quality of the cRNA samples were assessed by measuring the UV absorbance at 260 nm with a spectrophotometer and performing an electropherogram with 2100 Bioanalyzer (Agilent Technology), respectively. After fragmentation, the cRNA samples were hybridized on the slides (Human Whole Genome Oligonucleotides Microarrays 4×44k, G4112F, Agilent Technology) by using the Gene expression Hybridization kit (Agilent Technology). Arrays were then scanned (G2505R scanner, Agilent Technology) allowing simultaneous reading of the Cy3 and Cy5 fluorophores. The resolution was 5 mm and the settings were PMT cy3 XDR High 100%, Low 10% and PMT cy5 XDR High 100%, Low 10%. Image analysis was performed with Feature Extraction 9.1.3 software (Agilent Technology), following the manufacturer's recommendations (normalization: linear lowess). The software calculates a p-value giving the statistical significance on the LogRatio per each gene between the red and green channels. A gene is considered differentially expressed between two conditions if the difference is more than 2 fold and if the *P*value is less than 0.01 for all the replicates. Our data from the microarray analysis are deposited in the GEO repository (accession number GSE18875).

### RNA analysis by qRT-PCR

Total RNA was isolated using TRIZOL™LS reagent (Invitrogen). Quantity of the total RNA samples was assessed by measuring the UV absorbance at 260 and 280 nm with a spectrophotometer. Total RNA was reverse-transcribed using the iScript cDNA Synthesis kit (Biorad). Real-time quantitative PCR was carried out with the MyiQ Biorad Instrument using the iQ SYBR green Supermix for detection of PCR products (Biorad), according to the manufacturer's guidelines. Final concentrations of MgCl_2_ and primers were 3 mM and 0.3 µM, respectively. The sequences of the PCR primers and the size of amplicons are shown in Supporting Information S1. Care was taken to choose these oligonucleotides in different exons of the respective genes.

### Electromobility Shift Assays (EMSAs)

The RORα1 protein was obtained *in vitro* from the pCMX-hRORα1 vector using the TNT-T7 Quick coupled transcription/translation kit (Promega). DNA-protein complexes were allowed to form by incubating 1 µl of the programmed lysate for 20 min on ice in a 15 µl reaction containing 15 mM Tris-HCl, pH 7.5, 50 mM KCl, 15 mM NaCl, 1 mM MgCl_2_, 0.03 mM EDTA, 1 mM dithiothreitol, 7% glycerol (v/v), 50 ng of poly(dI-dC), 50 ng of unspecific single-stranded oligonucleotides and 0.15 ng (10,000 cpm) of the double-stranded oligonucleotide labeled by fill-in with the Klenow polymerase and [α-32-P]-dATP (3000 Ci/mmole, Amersham). For competition experiments, double-stranded oligonucleotides were added simultaneously with the labeled probe. Electrophoresis was performed at 200 V for 2 h at 15°C in 0.25× TBE buffer on a native 6% polyacrylamide gel that had been pre-run for 2 h under the same conditions. The gels were then fixed for 20 min in 20% ethanol, 10% acetic acid, dried and submitted to autoradiography.

### Chromatin Immunoprecipitation (ChIP)

ChIP was performed as described [52] with some modifications. Briefly, HepG2 cells were grown to 60% confluence. Cell lysates corresponding to 50×10^6^ cells were sonicated on ice 8 times for 1 min each. The sample was divided in two: one half was immunoprecipitated overnight at 4°C using ProteinA Ceramic HyperD®F (Pall) and 4 µg of goat anti-RORα antibody (Santa Cruz Biotechnology, Inc), and the other half was immunoprecipitated overnight at 4°C using ProteinA Ceramic HyperD®F (Pall) and 4 µg of normal goat IgG (Santa Cruz Biotechnology, Inc). One-sixtieth of the immunoprecipitated DNA was analyzed by real-time quantitative PCR on an ABI Prism 7500 apparatus using the Absolute QPCR SYBR Green ROX Mix (Thermo Scientific), according to the manufacturer's guidelines. Primers specific for the genomic regions containing ROREs identified *in silico* in *SPARC, PLG, G6PC, NR1D2 and AGRP* or specific for a genomic region located in *SPARC* and devoid of RORE (*SPARCn*) were used. Final concentrations of MgCl_2_ and primers were 3 mM and 0.3 µM, respectively. The sequences of the PCR primers and the size of amplicons are shown in Supporting Information S1.

### Sequence analysis

For each gene analyzed, the human regulatory regions (promoter or intragenic region) were extracted from sequence databases (Genbank; UCSC; ElDorado genome database using the Gene2Promoter software (Genomatix.de)). The promoter sequences were numbered relatively to the Transcription Start Site (TSS). In the case of multiple possible TSS we chose the more downstream TSS to determine the position of the putative RORE. The putative ROREs (close to the (A/T rich)_6_PuGGTCA consensus sequence) were selected by the MatInspector software (Genomatix.de) with regard to the 8 validated weight matrices for RORα or by manual search. For each regulatory region containing a putative RORE, we searched the conservation of the putative RORE in the orthologous sequences available in the ElDorado genome database (among of list of 20 organisms) or in the UCSC database.

### Statistical analysis

qRT-PCR, transfection and ChIP results are expressed as means ± S.E.M. for the number of experiments indicated in each case. Statistical analysis was performed using the ANOVA test followed by the Scheffe *post-hoc* test (Statview F-4.5 and Analyse-it softwares). A *P*value of less than 0.05 (*), 0.01 (**) or 0.001 (***) indicated significance.

## Supporting Information

Supporting Information S1 **PCR primers used in the qRT-PCR experiments (Table).** Gene names, sequences of the forward and reverse PCR primers, size of the PCR amplicon and the corresponding Genbank accession number are shown. **qPCR primers used in the ChIP experiments (Table).** Gene names, sequences of the forward and reverse qPCR primers and size of the PCR amplicon are shown.
(DOC)

Supporting Information S2 **Micro-array analysis of the effects of a stable RORα over-expression on gene expression in HepG2 cells (Table).** Complementary Table to Table 1 showing all the sequences (sequences corresponding to known genes and sequences not assigned to a known gene) found either up- or down-regulated in response to RORα over-expression. HepG2 cells were stably transfected with the pCMX-hRORα1 expression vector or with the pCMX insertless vector as a control. Total RNAs were extracted and mRNA levels were compared between these two experimental conditions with Agilent micro-arrays (n = 3 independent pools of transfected cells). This analysis revealed several genes whose expression was up-regulated or down-regulated by at least 2 fold in cells overexpressing RORα as compared to control cells. The gene symbols, gene description, genbank accession numbers and feature numbers on micro-arrays are given in columns 1 to 4. Gene expression levels in RORα over-expressing cells are expressed relatively to those in control cells. Results are given as fold changes (columns 5, 7, 9) and corresponding *P*values (columns 6, 8, 10). The means and S.E.M. of the fold changes are given in columns 11 and 12. All *P*values are less than 0.01 except one over three for *AGRP*.
(PDF)

Supporting Information S3 **Conservation in different species of the ROREs identified in the human genes (Table).** Analysis of the ROREs was performed as described in Materials and Methods. The gene symbols and the species are given in column 1. In the case of genes whose regulatory sequences contain two putative ROREs the gene symbol is followed by the number of the RORE (−1 or −2). Sequences containing the putative ROREs (in bold) are in column 2.
(DOC)
