# Supplementary material for: Control of Gene Expression by the Retinoic Acid-Related Orphan Receptor Alpha in HepG2 Human Hepatoma Cells
Source: PLoS One. 2011 Jul 26;6(7):e22545. doi: 10.1371/journal.pone.0022545 (PMC3144224; doi:10.1371/journal.pone.0022545)
Supplement: Supporting Information S1 — PCR primers used in the qRT-PCR experiments (Table). Gene names, sequences of the forward and reverse PCR primers, size of the PCR amplicon and the corresponding Genbank accession number are shown. qPCR primers used in the ChIP experiments (Table). Gene names, sequences of the forward and reverse qPCR primers and size of the PCR amplicon are shown. (DOC) [file pone.0022545.s001.doc]

**Supporting Information S1**

**Control of gene expression by the Retinoic acid-related Orphan Receptor alpha in HepG2 human hepatoma cells**

**Caroline CHAUVET^*‡^, Amandine VANHOUTTEGHEM^§^, , Christian DUHEM^¶^, Gaëlle SAINT-AURET^||^, Brigitte BOIS-JOYEUX^‡^, Philippe DJIAN^§^, Bart STAELS^¶^ and Jean-Louis DANAN^‡§^**

^*^Laboratoire de Pharmacologie, Toxicologie et Signalisation Cellulaire, INSERM UMR-S-747, Centre Universitaire des Saints Pères, Université Paris Descartes, 45 rue des Saints Pères, 75006 Paris, France

**^‡^**CNRS FRE-3210, Centre Necker, Université Paris Descartes, 156 rue de Vaugirard, 75015 Paris, France

^§^CNRS FRE-3235, Centre Universitaire des Saints Pères, Université Paris Descartes, 45 rue des Saints Pères, 75006 Paris, France

**^¶^**Université Lille Nord de France, Lille, France ; INSERM, U1011, Lille, France ; UDSL, Lille, France ; Institut Pasteur de Lille, Lille, France

**^||^**Faculté de Médecine et de Pharmacie, INSERM U-905, 22 boulevard Gambetta, 76183 Rouen, France

**Corresponding author:** Caroline Chauvet, PhD, Laboratoire de Pharmacologie, Toxicologie et Signalisation Cellulaire, INSERM UMR-S-747, Centre Universitaire des Saints Pères, Université Paris Descartes, Paris, France. Tel.: 33-1-42863864, Fax: 33-1-42863868, E-mail: caroline.chauvet@parisdescartes.fr

**PCR primers used in the qRT-PCR experiments**

Gene names, sequences of the forward and reverse PCR primers, size of the PCR amplicon and the corresponding Genbank accession number are shown.

| Gene name | PCR primer | Sequence (5’-3’) | Amplicon size (bp) | Genbank number |
| --- | --- | --- | --- | --- |
| *h36B4* | Forward | TCGACAATGGCAGCATCTAC | 223 | NM_001002 |
|  | Reverse | GCCTTGACCTTTTCAGCAAG |  |  |
| *hRora* | Forward | CTTCTTTCCCTACTGTTCGTTC | 128 | NM_134261 |
|  | Reverse | GCTCTTCTCTCAAGTATTGGC |  |  |
| *hSPARC* | Forward | GTGCAGAGGAAACCGAAGAG | 172 | NM_003118 |
|  | Reverse | TCATTGCTGCACACCTTCTC |  |  |
| *hPLG* | Forward | GTTTGGGAATGGGAAAGGAT | 186 | NM_000301 |
|  | Reverse | TAGCACCAGGGACCACCTAC |  |  |
| *hG6PC* | Forward | GAGACTGGCTCAACCTCGTC | 231 | NM_000151 |
|  | Reverse | TCGGCTTTATCTTTCCCTGA |  |  |
| *hRev-erbb* (*NR1D2*) | Forward | CAGCAATGTCGCTTCAAAAA | 124 | NM_005126 |
|  | Reverse | TGGTCTTCATTGCACTTTGC |  |  |
| *hAGRP* | Forward | CAACTGCAGAACAGGCAGAA | 191 | NM_001138 |
|  | Reverse | GCAGAAGGCATTGAAGAAGC |  |  |
| *hRev-erba* (*NR1D1*) | Forward | GACATGACGACCCTGGACTC | 123 | NM_021724 |
|  | Reverse | GCTGCCATTGGAGTTGTCAC |  |  |

**qPCR primers used in the ChIP experiments**

Gene names, sequences of the forward and reverse qPCR primers and size of the PCR amplicon are shown.

| Gene name | PCR primer | Sequence (5’-3’) | Amplicon size (bp) |
| --- | --- | --- | --- |
| *hSPARC* | Forward | TTGGGCCTGGTTCTGCCCCT | 154 |
|  | Reverse | CCGGGGCTGCTGCCTAAACC |  |
| *hPLG* | Forward | CTGCTGAGCCAGTGGCATGGGTC | 150 |
|  | Reverse | TGTTCCATTTTGGGACTGGCCAGC |  |
| *hG6PC* | Forward | GCCGATCAGGCTGTTTTTGTGTGC | 197 |
|  | Reverse | TGAGTCTGTGCCTTGCCCCTGT |  |
| *hNR1D2* | Forward | ACTCCAGCAGTGAAAGAAGTGGTGG | 114 |
|  | Reverse | AAAACCTACCTCAAAAGTCCCAGCC |  |
| *hAGRP* | Forward | CCATTGGCCCATCACCCGCT | 133 |
|  | Reverse | AAGGCAGGCGGCCCTGAAAG |  |
| *hSPARCn* | Forward | AAGGACCGAGGCTGCCACTTCAA | 177 |
|  | Reverse | GTGGTGGGAGGTAACCCGGCAT |  |
