# Supplementary material for: Control of Gene Expression by the Retinoic Acid-Related Orphan Receptor Alpha in HepG2 Human Hepatoma Cells
Source: PLoS One. 2011 Jul 26;6(7):e22545. doi: 10.1371/journal.pone.0022545 (PMC3144224; doi:10.1371/journal.pone.0022545)
Supplement: Supporting Information S2 — Micro-array analysis of the effects of a stable RORα over-expression on gene expression in HepG2 cells (Table). Complementary Table to Table 1 showing all the sequences (sequences corresponding to known genes and sequences not assigned to a known gene) found either up- or down-regulated in response to RORα over-expression. HepG2 cells were stably transfected with the pCMX-hRORα1 expression vector or with the pCMX insertless vector as a control. Total RNAs were extracted and mRNA levels were compared between these two experimental conditions with Agilent micro-arrays (n = 3 independent pools of transfected cells). This analysis revealed several genes whose expression was up-regulated or down-regulated by at least 2 fold in cells overexpressing RORα as compared to control cells. The gene symbols, gene description, genbank accession numbers and feature numbers on micro-arrays are given in columns 1 to 4. Gene expression levels in RORα over-expressing cells are expressed relatively to those in control cells. Results are given as fold changes (columns 5, 7, 9) and corresponding Pvalues (columns 6, 8, 10). The means and S.E.M. of the fold changes are given in columns 11 and 12. All Pvalues are less than 0.01 except one over three for AGRP. (PDF) [file pone.0022545.s002.pdf]

Supporting Information S2

Control of gene expression by the Retinoic acid-related Orphan Receptor alpha in HepG2 human hepatoma cells

Caroline CHAUVET[*‡], Amandine VANHOUTTEGHEM[§], , Christian DUHEM[¶], Gaëlle SAINT-AURET[‖], Brigitte BOIS-JOYEUX[‡], Philippe DJIAN[§], Bart STAELS[¶] and Jean-Louis DANAN[‡§]

[*]Laboratoire de Pharmacologie, Toxicologie et Signalisation Cellulaire, INSERM UMR-S-747, Centre Universitaire des Saints Pères, Université Paris Descartes, 45 rue des Saints Pères, 75006 Paris, France
[‡]CNRS FRE-3210, Centre Necker, Université Paris Descartes, 156 rue de Vaugirard, 75015 Paris, France
[§]CNRS FRE-3235, Centre Universitaire des Saints Pères, Université Paris Descartes, 45 rue des Saints Pères, 75006 Paris, France
[¶]Université Lille Nord de France, Lille, France ; INSERM, U1011, Lille, France ; UDSL, Lille, France ; Institut Pasteur de Lille, Lille, France
[‖]Faculté de Médecine et de Pharmacie, INSERM U-905, 22 boulevard Gambetta, 76183 Rouen, France

Corresponding author: Caroline Chauvet, PhD, Laboratoire de Pharmacologie, Toxicologie et Signalisation Cellulaire, INSERM UMR-S-747, Centre Universitaire des Saint s Pères, Université Paris Descartes, Paris, France. Tel.: 33-1-42863864, Fax: 33-1-42863868, E-mail: caroline.chauvet@parisdescartes.fr

Micro-array analysis of the effects of a stable RORα over-expression on gene expression in HepG2 cells.
Complementary Table to Table 1 showing all the sequences (sequences corresponding to known genes and sequences not assigned to a known gene) found either up- or down-regulated in response to RORα over-expression.
HepG2 cells were stably transfected with the pCMX-hRORα1 expression vector or with the pCMX insertless vector as a control. Total RNAs were extracted and mRNA levels were compared between these two experimental conditions with Agilent micro-arrays (n = 3 independent pools of transfected cells). This analysis revealed several genes whose expression was up-regulated or down-regulated by at least 2 fold in cells overexpressing RORα than in control cells. The gene symbols, gene description, genbank accession numbers and feature numbers on micro-arrays are given in columns 1 to 4. Gene expression levels in RORα over-expressing cells are expressed relatively to those in control cells. Results are given as fold changes (columns 5, 7, 9) and corresponding *P*values (columns 6, 8, 10). The means and S.E.M. of the fold changes are given in columns 11 and 12. All *P*values are less than 0.01 except one over three for *AGRP*.

Fold more than 2, p-value less than 0,01 for all the 3 replicates (except for one AGRP replicate)

GeneSymbol	Gene description	Genbank accession	Feature Number	fold 1	PValueLogRatio 1	fold 1	PValueLogRatio 2	fold 3	PValueLogRatio 3	Mean	S.E.M.
UP-REGULATED											
PLG	Homo sapiens plasminogen (PLG), mRNA [NM_000301]	NM_000301	12618	25,8350	6,3981E-15	27,2424	1,3905E-19	29,6528	2,1463E-19	27,5768	1,11
PLG	Homo sapiens plasminogen (PLG), mRNA [NM_000301]	NM_000301	31563	9,1322	1,4887E-12	20,9459	2,0886E-18	24,1585	8,8075E-19	18,0789	4,57
RORA	Homo sapiens RAR-related orphan receptor A (RORA), transcript variant 2, mRNA [NM_134260]	NM_134260	31288	12,9788	3,7275E-20	16,4481	7,2882E-21	13,8347	2,3283E-20	14,4205	1,04
LPA	Homo sapiens lipoprotein, Lp(a) (LPA), mRNA [NM_005577]	NM_005577	31776	10,0751	3,5886E-12	8,4043	1,0202E-14	9,5955	2,4666E-16	9,3583	0,50
FBXL17	Homo sapiens F-box and leucine-rich repeat protein 17, mRNA (cDNA clone IMAGE:4215262), partial cds. [BC018548]	BC018548	391	5,4904	1,1319E-08	2,6927	9,2040E-05	2,1936	4,8845E-05	3,4589	1,03
G6PC	Homo sapiens glucose-6-phosphatase, catalytic subunit (G6PC), mRNA [NM_000151]	NM_000151	19594	2,7782	6,7296E-07	3,0260	4,0621E-07	2,6103	3,2640E-06	2,8048	0,12
SPARC	Homo sapiens secreted protein, acidic, cysteine-rich (osteonectin) (SPARC), mRNA [NM_003118]	NM_003118	4259	2,8443	1,1311E-09	2,2330	5,1936E-07	2,0311	5,6378E-06	2,3695	0,24
AGRP	Homo sapiens agouti related protein homolog (mouse) (AGRP), transcript variant 1, mRNA [NM_001138]	NM_001138	21543	1,4368	1,3359E-01	2,7437	1,8983E-03	2,6587	6,1903E-04	2,2797	0,42
	Q8PBG7 (Q8PBG7) Riboflavin biosynthesis protein, partial (6%) [THC2317871]	THC2317871	39638	2,1643	3,2376E-04	2,4661	5,1446E-05	2,0864	7,1564E-05	2,2389	0,12
NR1D2	Homo sapiens nuclear receptor subfamily 1, group D, member 2, mRNA (cDNA clone IMAGE:3912370), partial cds. [BC015929]	BC015929	31530	2,0783	3,0420E-05	2,9059	2,0246E-08	1,6813	1,8611E-03	2,2219	0,36
SMOC1	Homo sapiens SPARC related modular calcium binding 1 (SMOC1), transcript variant 2, mRNA [NM_022137]	NM_022137	32907	2,1044	2,5417E-06	1,7507	2,0939E-04	2,6761	5,1054E-09	2,1770	0,27
HEPN1	Homo sapiens associated with liver cancer (HEPN1), mRNA [NM_001037558]	NM_001037558	34970	1,8068	2,5819E-03	2,1847	1,5170E-05	2,4995	9,6517E-07	2,1636	0,20
FOXO4 (MLLT7)	Homo sapiens myeloid/lymphoid or mixed-lineage leukemia (trithorax homolog, Drosophila); translocated to, 7 (MLLT7), mRNA [NM_005938]	NM_005938	42837	1,9082	4,5203E-03	1,9981	1,0212E-03	2,2200	9,1436E-05	2,0421	0,09
DOWN-REGULATED											
	Homo sapiens clone FLB9714 PRO2619 mRNA, complete cds. [AF130077]	AF130077	41972	0,5690	2,0520E-04	0,4813	3,9126E-06	0,4471	5,3386E-07	0,4991	0,04
	Unknown	THC2290704	17190	0,4770	5,1612E-03	0,5837	9,6946E-03	0,4018	3,3592E-04	0,4875	0,05
	AA906057 oj90d07.s1 Soares_NFL_T_GBC_S1 Homo sapiens cDNA clone IMAGE:1505581 3', mRNA sequence [AA906057]	AA906057	12231	0,3960	1,3178E-04	0,5552	6,6930E-03	0,4593	1,2755E-04	0,4701	0,05
	Homo sapiens full length insert cDNA clone ZD54C08. [AF086329]	AF086329	24011	0,4937	9,0537E-06	0,4160	1,4739E-07	0,5004	1,1650E-05	0,4700	0,03
	UI-H-FH1-bfk-o-16-0-UI.s1 NCI_CGAP_FH1 Homo sapiens cDNA clone UI-H-FH1-bfk-o-16-0-UI 3', mRNA sequence [BU618641]	BU618641	8692	0,3221	1,2861E-06	0,4774	2,4852E-04	0,6019	3,2313E-03	0,4671	0,08
	Unknown	A_32_P45087	1685	0,5485	1,2095E-02	0,3378	6,2418E-06	0,4970	1,4128E-03	0,4611	0,06
	ze57f04.s1 Soares retina N2b4HR Homo sapiens cDNA clone IMAGE:363103 3' similar to gb:X62534 HIGH MOBILITY GROUP PROTEIN HMG2 (HU	AA019203	42520	0,4838	7,8389E-03	0,4019	8,6242E-05	0,4912	9,8583E-04	0,4590	0,03
	AI919230 qy30h02.x1 NCI_CGAP_Brn23 Homo sapiens cDNA clone IMAGE:2013555 3', mRNA sequence [AI919230]	AI919230	34849	0,4073	1,8662E-04	0,5321	1,6148E-03	0,4110	5,2675E-05	0,4502	0,04
	BX419477 Homo sapiens FETAL BRAIN Homo sapiens cDNA clone CS0DF017YM02 3-PRIME, mRNA sequence [BX419477]	BX419477	6517	0,4720	2,1893E-03	0,4411	2,4812E-04	0,4199	1,7249E-05	0,4443	0,02
	Unknown	A_32_P20014	777	0,2931	9,0720E-08	0,4402	1,7503E-05	0,5817	7,9199E-03	0,4384	0,08
	BE749146 601123310F1 NIH_MGC_5 Homo sapiens cDNA clone IMAGE:3348051 5', mRNA sequence [BE749146]	BE749146	18408	0,4756	2,0711E-03	0,3673	1,9552E-04	0,4549	3,9193E-04	0,4326	0,03
	BE719776 RC3-HT0865-260700-011-b02 HT0865 Homo sapiens cDNA, mRNA sequence [BE719776]	BE719776	28582	0,3165	1,1636E-05	0,4731	4,6521E-04	0,3754	1,1011E-06	0,3883	0,05
	Unknown	A_32_P135790	21089	0,3839	1,2654E-07	0,4256	1,7949E-06	0,2813	2,6318E-11	0,3636	0,04
LGI2	Homo sapiens leucine-rich repeat LGI family, member 2 (LGI2), mRNA [NM_018176]	NM_018176	24947	0,1917	1,8768E-05	0,3630	4,5701E-04	0,3684	2,4577E-03	0,3077	0,06
ADIPOQ	Homo sapiens adiponectin, C1Q and collagen domain containing (ADIPOQ), mRNA [NM_004797]	NM_004797	36194	0,3411	3,2106E-03	0,1707	6,5835E-04	0,1700	4,5097E-04	0,2272	0,06
