# Supplementary material for: Control of Gene Expression by the Retinoic Acid-Related Orphan Receptor Alpha in HepG2 Human Hepatoma Cells
Source: PLoS One. 2011 Jul 26;6(7):e22545. doi: 10.1371/journal.pone.0022545 (PMC3144224; doi:10.1371/journal.pone.0022545)
Supplement: Supporting Information S3 — Conservation in different species of the ROREs identified in the human genes (Table). Analysis of the ROREs was performed as described in Materials and Methods. The gene symbols and the species are given in column 1. In the case of genes whose regulatory sequences contain two putative ROREs the gene symbol is followed by the number of the RORE (−1 or −2). Sequences containing the putative ROREs (in bold) are in column 2. (DOC) [file pone.0022545.s003.doc]

**Supporting Information S3**

**Control of gene expression by the Retinoic acid-related Orphan Receptor alpha in HepG2 human hepatoma cells**

**Caroline CHAUVET^*‡^, Amandine VANHOUTTEGHEM^§^, , Christian DUHEM^¶^, Gaëlle SAINT-AURET^||^, Brigitte BOIS-JOYEUX^‡^, Philippe DJIAN^§^, Bart STAELS^¶^ and Jean-Louis DANAN^‡§^**

^*^Laboratoire de Pharmacologie, Toxicologie et Signalisation Cellulaire, INSERM UMR-S-747, Centre Universitaire des Saints Pères, Université Paris Descartes, 45 rue des Saints Pères, 75006 Paris, France

**^‡^**CNRS FRE-3210, Centre Necker, Université Paris Descartes, 156 rue de Vaugirard, 75015 Paris, France

^§^CNRS FRE-3235, Centre Universitaire des Saints Pères, Université Paris Descartes, 45 rue des Saints Pères, 75006 Paris, France

**^¶^**Université Lille Nord de France, Lille, France ; INSERM, U1011, Lille, France ; UDSL, Lille, France ; Institut Pasteur de Lille, Lille, France

**^||^**Faculté de Médecine et de Pharmacie, INSERM U-905, 22 boulevard Gambetta, 76183 Rouen, France

**Corresponding author:** Caroline Chauvet, PhD, Laboratoire de Pharmacologie, Toxicologie et Signalisation Cellulaire, INSERM UMR-S-747, Centre Universitaire des Saints Pères, Université Paris Descartes, Paris, France. Tel.: 33-1-42863864, Fax: 33-1-42863868, E-mail: caroline.chauvet@parisdescartes.fr

**Conservation in different species of the ROREs identified in the human genes**

Analysis of the ROREs was performed as described in Materials and Methods.

The gene symbols and the species are given in column 1. In the case of genes whose regulatory sequences contain two putative ROREs the gene symbol is followed by the number of the RORE (-1 or -2). Sequences containing the putative ROREs (in bold) are in column 2.
